# Differences in Vaccination Consultation Preferred by Primary Health Care Workers and Residents in Community Settings

**DOI:** 10.3390/vaccines12050534

**Published:** 2024-05-14

**Authors:** Tianshuo Zhao, Xianming Cai, Sihui Zhang, Mingting Wang, Linyi Chen, Juan Wang, Yajie Yu, Liandi Tao, Xiaoxia Xu, Jing Luo, Chao Wang, Juan Du, Yaqiong Liu, Qingbin Lu, Fuqiang Cui

**Affiliations:** 1 Department of Laboratorial Science and Technology & Vaccine Research Center, School of Public Health, Peking University, Beijing 100191, China; 2 Center for Infectious Diseases and Policy Research & Global Health and Infectious Diseases Group, Peking University, Beijing 100191, China; 3 Key Laboratory of Epidemiology of Major Diseases (Peking University), Ministry of Education, Beijing 100191, China; 4 Department of Epidemiology, School of Public Health, University of Pittsburgh, Pittsburgh, PA 15260, USA; 5 Jiuzhaigou Center for Disease Control and Prevention, Ngawa 623099, China; 18909044897@163.com; 6 Yilan Center for Disease Control and Prevention, Harbin 154899, China; 7 Longxi Center for Disease Control and Prevention, Longxi 748199, China; 8 Chengguan Center for Disease Control and Prevention, Lanzhou 730030, China; 18193209677@163.com; 9 Suzhou Center for Disease Control and Prevention, Suzhou 234099, China

**Keywords:** vaccination consultation, vaccine hesitancy, primary health care workers, community residents

## Abstract

Objective: To evaluate the preference of primary HCWs and residents on vaccination consultation in community health services to provide evidence for vaccine hesitancy intervention strategies. Methods: A discrete choice model (DCM) was constructed to evaluate the preference difference between primary HCWs and residents on vaccination consultation in community health services in China during May–July 2022. Results: A total of 282 residents and 204 HCWs were enrolled in this study. The residents preferred consulting with an HCW-led approach (β = 2.168), with specialized content (β = 0.954), and accompanied by telephone follow-up (β = 1.552). In contrast, the HCWs preferred face-to-face consultation (β = 0.540) with an HCW-led approach (β = 0.458) and specialized content (β = 0.409), accompanied by telephone follow-up (β = 0.831). College residents and residents with underlying self-reported disease may be near-critically inclined to choose traditional consultation (an offline, face-to-face consultation with standardized content and more prolonged duration) rather than a new-media consulting group (an online consultation with specialized content within 5 min). Urban HCWs preferred long-term consultation groups (the resident-led offline consultation with follow-up lasting more than 5 min). In contrast, rural HCWs preferred efficient consultation (the HCW-led, short-duration, standardized offline consultation mode). Conclusion: The selection preference for vaccine consultation reveals a gap between providers and demanders, with different groups exhibiting distinct preferences. Identifying these targeted gaps can help design more acceptable and efficient interventions, increasing their likelihood of success and leading to better resource allocation for policymakers to develop targeted vaccination policies.

## 1. Introduction

The World Health Organization (WHO) has defined vaccine hesitancy as refusing or postponing vaccination even when vaccine services are accessible. Factors influencing vaccine hesitancy vary over time, place, availability, trust, complacency, and convenience [1]. In 2019, the WHO confirmed vaccine hesitancy as one of the top ten global threats due to its indirect hindrance to preventing and controlling infectious diseases through impacting vaccine coverage. The accumulation of widespread hesitancy behaviors may even result in the resurgence and outbreak of pathogens. Vaccine hesitancy poses a long-term global challenge that has gained particular significance during the COVID-19 pandemic, with varying levels of vaccine acceptance observed worldwide. This is influenced by numerous factors, especially in the context of extensive internet usage, where misinformation about vaccines inundates social media platforms and specific news channels, leading individuals to question vaccines’ safety and efficacy while failing to accurately perceive their vaccination needs. Increasing the popularization of vaccine-related healthcare information through multiple channels is also key to reducing vaccine hesitancy [2].

Scientific information helps the population develop a positive awareness and attitude around vaccination. Thus, vaccination consultation is widely recognized as one of the most potent measures for eliminating vaccine hesitancy in the general population [3]. Primary health care workers (HCWs) play a vital role in maintaining the health of the population because of the continuity of vaccination-related services they provide [4]. Vaccination advice given by HCWs has a predominant impact on people’s vaccination decisions [5,6]. The existing literature often discusses interviewing skills from the physician’s perspective through qualitative interviews and analyzes vaccination confidence from the patient’s perspective using descriptive methods. What is needed now is a strategy that combines the perspectives of both HCWs and residents (namely suppliers and demanders in vaccination consulting) to analyze consultation strategies from the perspective of policymakers.

Thus, this research aims to examine the complex nature of both perspectives by combining surveys from residents and HCWs to account for the fact that vaccination is a healthcare service that HCWs provide and residents demand. The interaction between supply and demand is crucial for ensuring effective vaccination programs but discrepancies may exist between HCWs’ beliefs on consultation approaches and residents’ preferences [7]. Identifying these gaps can help design more acceptable interventions for residents, increasing their likelihood of success [8]. By surveying HCWs and residents, this study aimed to bridge understanding gaps in divergent preferences, leading to better resource allocation for policymakers to develop targeted vaccination policies.

The discrete choice model (DCM) provides a convenient way to interpret or predict choices from a set of discrete alternatives [9]. In healthcare, DCM can simulate choice environments for different health services, measuring preferences across diverse populations [10]. DCM excels in revealing behavioral patterns, offering a more effective quantification of preferences than typical choice questions. One can identify influential factors and their impact on behavior by analyzing estimated values. Another key feature is its capacity for simulation analysis. Unlike traditional scenarios where respondents do not consider additional factors, DCM explores potential differences resulting from changes in various factors and provides a forward-looking assessment [11,12,13].

Based on the abovementioned research, we postulate that divergent preferences for vaccination consultation exist between HCWs and residents, who serve as the suppliers and demanders of community health services, with different groups exhibiting distinct preferences. This study used DCM to understand community residents’ preferences for vaccination consultation services to identify the optimal combination of factors as evidence for informed intervention strategy decisions. Therefore, a DCM was constructed to evaluate the preference of primary HCWs and residents for vaccination consultation in community health services and provide evidence for vaccine hesitancy intervention strategies.

## 2. Materials and Methods

### 2.1. Enrollment

Two survey sites, Rencheng District and Jinxiang County, were set up in Jining, Shandong Province of China, from May 2022 to July 2022. The convenience sampling method was adopted to recruit HCWs and residents who worked in the community health centers or lived in the communities affiliated with the two sites. China is a developing nation situated in the Western Pacific region. This study chose a city with moderate economic and cultural development in eastern China. Considering the presence of distinct dialects, particularly in rural areas, local individuals affiliated with the regional Center for Disease Control and Prevention were engaged to facilitate field implementation.

The criteria of the resident subjects: (a) at least 18 years old; (b) permanent residents living in the community; (c) having normal reading and understanding abilities and having the ability to share details about independent behavior; (d) informed consent and voluntary participation in this study.

The criteria of the HCWs: (a) at least 18 years old; (b) HCWs in a community health center; (c) having normal reading and understanding ability and having the ability to share details about independent behavior; (d) informed consent and voluntary participation in this study.

This study established survey sites in the local community, with the community health service center assuming responsibility for promoting and enrolling participants for this research project. Subjects that met the criteria were invited to fill out an online questionnaire supported by the Changsha Ranxing Information Technology Co. (Changsha, China), a platform supporting the online collection and storage of data. WeChat, a popular instant messaging program widely used in China (WeChat, Copyright 1998–2023 Tencent, Shenzhen, China), distributed questionnaires constructed by Ranxing for the survey. By scanning the two-dimensional code on promotional materials for WeChat or clicking the link in the community’s WeChat group, community members could complete the electronic questionnaire.

### 2.2. Questionnaire

The questionnaire had two parts (see Supplementary S1 and S2). The first was a sociodemographic survey that included age, sex, educational level, monthly income, and self-reported health status for residents, as well as work age, workplace, and professional title. The self-reported health status pertains to the residents’ self-diagnosis of any of the following conditions: obesity (BMI > 30), hypertension, hyperlipidemia, diabetes, cardiovascular and cerebrovascular diseases, chronic lung diseases, chronic liver diseases, chronic kidney disease or neoplasm, tumor, immune dysfunction, or other underlying ailments. Work age is defined as the duration of employment for HCWs with wage income as their primary or sole source since establishing labor relations with the organization. We classify HCWs’ workplaces as either rural or urban by considering whether they work in an urban community health service center or a rural township health center. Professional titles are designations conferred by the Chinese government’s personnel and health departments to healthcare workers (HCWs) based on their level of professional expertise, categorized as junior (e.g., medical assistant/physician), intermediate (e.g., attending physician), and senior (e.g., associate chief physician and chief physician).

The second part is the discrete choice experiment (DCE), which is designed based on the process of community vaccination consultation behavior. Five attributes were constructed for the DCE, and two levels were set for each attribute: 

(a) Consulting-led: to examine who initiated the consultation (HCWs or residents); 

(b) Consulting duration: defined as <5 min or ≥5 min on each vaccination consultation service; 

(c) Consulting content: A standardized consultation means that the HCWs provide vaccination advice to residents using fully standardized consultation guidelines. A specialized consultation means that it relies more on the subjective judgment of the HCW and the individual needs of the residents rather than the guidelines, and the consultation service provided each time is different from person to person; 

(d) Consulting mode: refers to the consultation through an online model such as new media or instant messaging, or face-to-face offline consultation; 

(e) Telephone follow-up: Whether regular follow-up of vaccination status or consulting demands is required after consultation. The follow-up involved assessing the impact of the previous vaccination consultation service on residents, including evaluating whether their needs were met during the last consultation, examining any changes in their vaccination behavior resulting from the last consultation, and identifying any new consultation requirements that may have arisen.

For each option set, two consulting options and one opt-out option were set, resulting in 2^5^ possible combinations of scenarios. To reduce the number of options for respondents, the selection set of different level combinations was optimized through partial factorial designs, including orthogonal designs and D-efficiency designs. Eight sets of choice scenarios were generated. An additional set of choice options was added to each questionnaire, and a random number method was used to select a duplicate set of options to test whether the choices were consistent.

### 2.3. Statistical Analysis

The sample size calculation formula for DCM is expressed as follows:

(1) $$N=\frac{500\times c}{t\times a}$$

where *c* is the maximum number of levels in the attribute, *t* represents the number of choice sets in each questionnaire, and *a* refers to the number of options in each choice set. In this questionnaire, *c* = 2, *t* = 2, and *a* = 5, the minimum sample size required for this study is therefore set at 100. Finally, the planned adequate sample size is established as 150, considering the need to increase the sample size of non-random sampling to 1.5–2 times that of random sampling. 

This study analyzed the experimental data based on the random utility maximization theory, using a mixed logit model and a latent class logit model. The mixed logit model analyzed residents’ consultation behavior preferences. The relative importance (RI) of each level is estimated by dividing the difference between the lowest and highest utility levels of the attribute by the sum of differences across all attribute levels. The latent class logit model was used to classify residents into different latent classes. Latent class conditional logit models were fitted through an Expectation–Maximization algorithm proposed by Bhat and Train [14,15]. The optimal number of latent classes and best model fit were determined based on the values of the Akaike information criterion (AIC) and the Bayesian information criterion (BIC), with the model having the lowest AIC and BIC values selected. The probability of choosing each alternative in the posterior probabilities of class membership was predicted, that the agent is in particular classes considering the sequence of choices. For each individual given the choice probabilities, the latent class in which they were predicted to choose with the most significant relative probability is identified as their latent grouping. Additionally, a non-conditional logistic model was constructed to explore the differences in demand preferences among latent groups. All analyses were conducted using Stata 17.0.

## 3. Results

### 3.1. Characteristics of Participants and the Consistency Test

A total of 282 residents and 204 HCWs were finally enrolled in this study (Table 1). Among the study subjects included, a total of 223 (79.08%) residents and 158 (77.45%) HCWs passed the consistency test. 

The consistency test showed that the direction of the regression coefficient was consistent between all subjects and passed subjects, indicating that the subjects that did not pass the consistency test did not significantly interfere with the regression results. However, the null hypothesis was rejected in the likelihood ratio test; that is, it could not prove that model 1 (model of all included subjects) was nested in model 2 (model of the subjects who passed the consistency test), indicating that the inconsistent subjects interfered with the model results. Therefore, only the sample size that passed the consistency test was used for the final analysis of consultation preferences in this study Supplementary S3 and S4).

### 3.2. The Mixed Logit Models of Vaccination Consultation from Residents and HCWs

**Figure 1:**
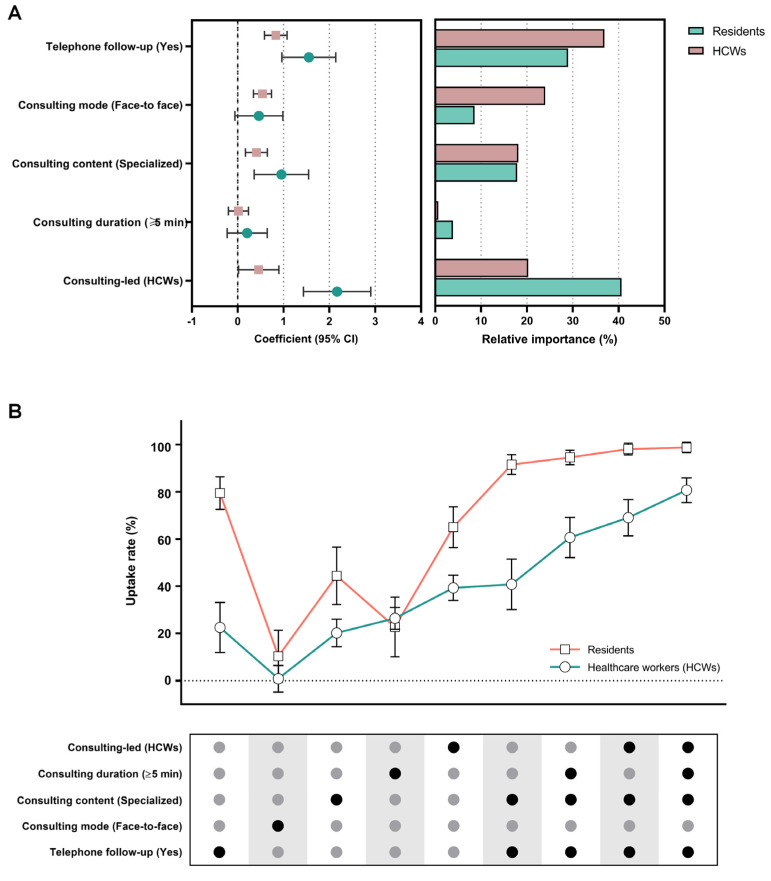
The vaccination counseling preference from residents and healthcare workers (HCWs). (**A**) The mixed logit model of vaccination counseling preference. (**B**) The uptake rate of vaccination consultation under different consulting scenarios among residents and healthcare workers (HCWs).

The results of the mixed logit model are shown in Figure 1A. The attributes of consultation-led, consultation content, and telephone follow-up significantly impact the consulting service preferences of the residents. The residents prefer consulting with an HCW-led approach (β = 2.168), with specialized content (β = 0.954), and accompanied by telephone follow-up (β = 1.552). For the residents, the predominant factor is consulting-led (40.6%), followed by telephone follow-up (29.0%), and consulting content (17.9%) (Figure 1A).

Similarly, the consulting preferences of HCWs were also influenced by consulting-led, consulting content, and telephone follow-up, in addition to consulting mode. The HCWs prefer face-to-face consultation (β = 0.540) with an HCW-led approach (β = 0.458) and specialized content (β = 0.409), accompanied by telephone follow-up (β = 0.831). For the supply side, the relative importance of consulting content is the highest (36.9%), followed by consulting-led (20.3%) and telephone follow-up (24.0%), as shown in Figure 1A.

The uptake rate was predicted to anticipate the change in the probability of choice for various consultation scenarios, with no follow-up, online mode, standardized content, duration of <5 min, and resident-led as the baseline scenario. When compared to the baseline, the uptake rate of HCWs increased by 39.3% in the scenario with HCW-led approaches, followed by the longer duration (26.4%), availability of telephone follow-up (22.5%), and specialized content (20.1%) (Figure 1B). For residents, changing the consultation scenarios to telephone follow-up (79.5%), specialized content (44.4%), over 5 min in length (22.7%), and HCW-led (65.1%), respectively, resulted in some change in the likelihood that the HCWs would choose consultation services (Figure 1B).

Afterward, several sets of practicable consulting scenarios were bonded to forecast the impact of different combinations of consulting services on contracting decisions. HCW-side consulting services in the optimal scenario for selecting consulting services, i.e., telephone follow-up, specialized content, and HCW-led consultations over 5 min, would increase HCWs’ probability of contracting consultation by 80.7% compared to the baseline level. For residents, the likelihood increase of 91.6% can be attained when the consultation service is improved with follow-up plus specialized content, with increases of 98.8% when consulting-led and consulting duration were added (Figure 1B).

### 3.3. The Latent Class Logit Model of Vaccination Consultation from HCWs and Residents

**Figure 2:**
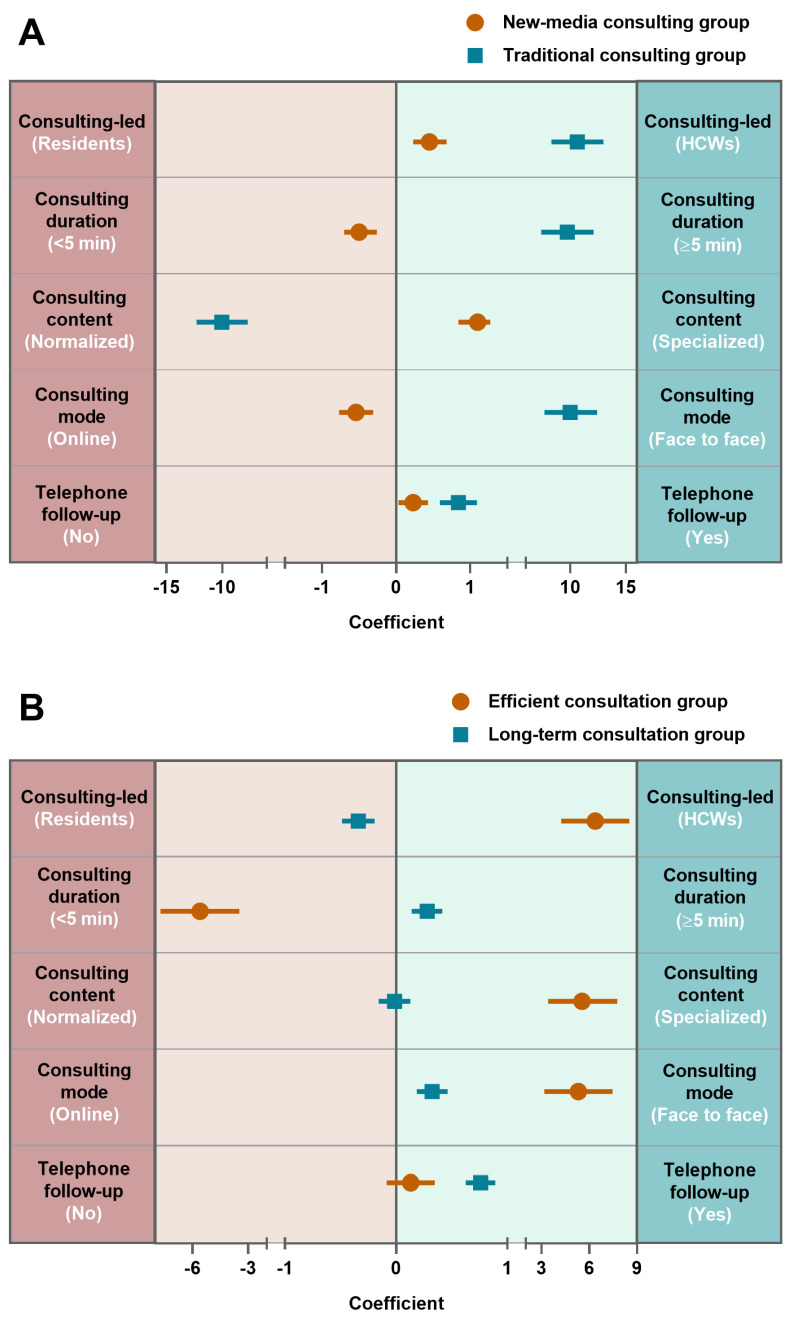
The binary latent class model results in the preference for vaccine consultation among residents and healthcare workers (HCWs). Coefficient 0 is the dividing line of selection preference. When the coefficient and confidence intervals are less than 0, the parameter on the left side tends to be selected. Otherwise, select the parameters on the right. (**A**) The latent class results in residents; (**B**) the latent class results in HCWs.

The latent logit model was used to develop resident models for two categories, and the results are displayed in Figure 2. The residents were grouped into two groups, the “traditional consulting group” and the “new-media consulting group”, according to their characteristics. The traditional consultation group, which can be characterized as an offline, face-to-face consultation with standardized content as a guideline and a longer duration (more than 5 min), was preferred by 109 residents (48.9%). There were 114 residents (51.1%) in the new-media consultation group who picked and chose online consultation with specialized content within 5 min. In addition, residents in both categories tended to prefer HCW-led consultations with telephone follow-up (Figure 2A). 

**Figure 3:**
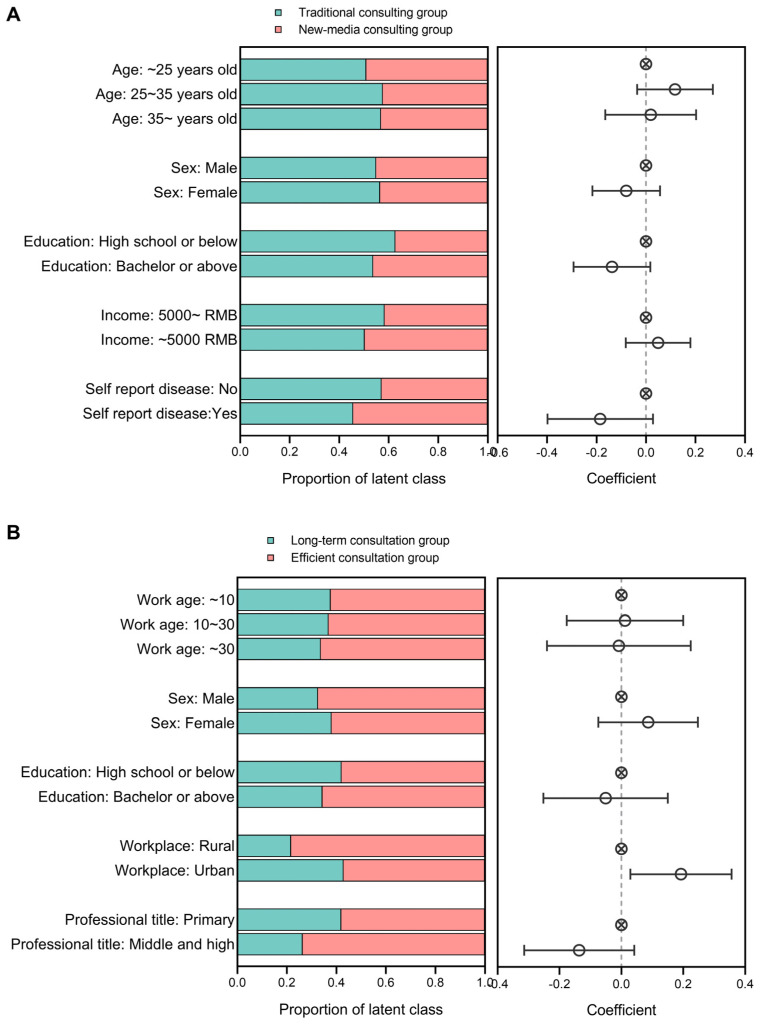
The logistic regression results in the sociodemographic difference between the two latent groups among residents and healthcare workers (HCWs). (**A**) The regression results in residents; (**B**) the regression results in HCWs.

The selection probability of traditional consultation was used as the dependent variable for regression analysis to examine the determining factors of preferences to investigate the differences between the two categories of people but no statistically significant characteristics were discovered. Nevertheless, two borderline results deserve more attention: resident groups with a bachelor and above education and underlying self-reported disease could be more inclined to choose traditional consultation, namely, standardized face-to-face consultation over a more extended period (Figure 3A).

For HCWs, 99 subjects (62.66%) preferred a “long-term consultation group” and favored the resident-led offline consultation, with follow-up lasting more than 5 min. In the category “efficient consultation group”, 59 HCWs (37.34%) were found who favored the HCW-led, short-duration, standardized offline consultation mode. Consequently, “long-term consultation” and “efficient consultation” were the names of the two recognized probable categories of provider consultation preferences (Figure 2B).

Regression analysis was employed to examine the differences in social demographic features between the two groups, with the selection probability of long-term follow-up consultation as the dependent variable. The results suggest that HCWs in urban areas were more inclined to choose long-term consultation, while HCWs in rural were more likely to opt for efficient consultation (Figure 3B).

**Figure 4:**
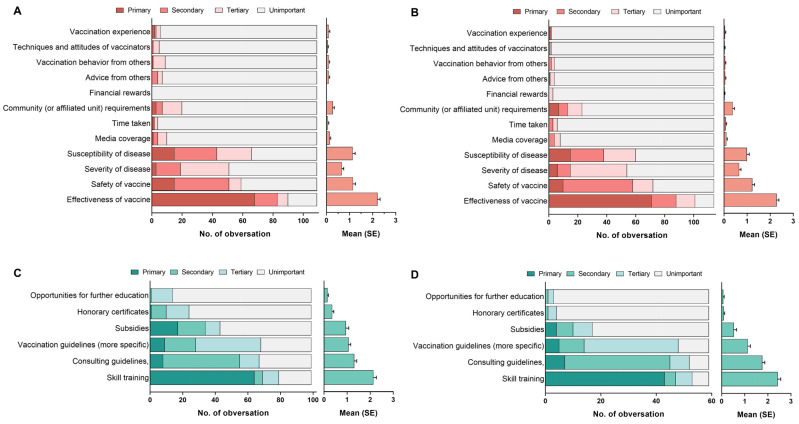
The vaccination decision motives and preferences of consulting improvements among residents and healthcare workers (HCWs). (**A**) The vaccination decision motives of traditional consulting group; (**B**) the vaccination decision motives of new-media consulting group; (**C**) the preference of consulting improvements of long-term consultation group; (**D**) the preference of consulting improvements of efficient consultation group.

Finally, an exploratory comparison was made between the vaccination decision motives of the two resident groups, while no statistical distinction was revealed (Figure 4A,B). The two HCW groups that preferred consulting improvements were compared, showing that HCWs who preferred efficient consultation were more likely to improve consulting guidelines than subsidies and honorary certificates (Figure 4C,D).

## 4. Discussion

### 4.1. Attributes of the Vaccination Consultation

The intrinsic factors that can enhance vaccination coverage are diverse, including trust in vaccines, trust in healthcare institutions, access to sufficient information on vaccination, high levels of health literacy, and effective communication and advice from HCWs [16,17,18,19]. All these factors can be explained as providing accurate knowledge and information to residents [20]. Doctors, including HCWs, are one of the most trusted sources of information for residents, and their recommendations play a crucial role in reducing vaccine hesitancy [3].

From the perspective of the health belief framework, when individuals are suitable for vaccination but are unwilling to vaccinate, they may misjudge the threat of disease and the pros and cons of vaccination [21,22]. Studies have shown that vaccination consultation significantly impacts individuals with lower perceptions of vaccine safety, supporting the explanation that consultation can reduce concerns about vaccine safety and further improve vaccine coverage [20]. Vaccination consultation can improve the perception of vaccine-related issues among residents, particularly regarding vaccine safety. This is mainly due to the increasing importance of vaccine safety concerns, and individuals’ trust in HCWs allows consultation to improve this negative perception [23,24].

This study explores ways to improve vaccination consultations based on the consultation process, including the initiator, residents, and the consultation itself. The initiation of consultation can come from patients actively seeking help from doctors or doctors proactively inquiring about the residents’ vaccination needs. Standardized consulting modes allow HCWs to provide vaccination recommendations to residents using standardized consulting guidelines, while specialized consulting modes focus more on the genuine needs of residents and promote interaction [25,26,27]. Traditional consultation often occurs in primary healthcare settings’ vaccination clinics; however, with the development of internet platforms and artificial intelligence technologies, residents can access desired information through social media platforms such as WeChat official accounts, which are widely used for information display and dissemination in China [28,29]. After residents have sought consultation, healthcare providers can follow up on their vaccination status periodically through smart voice calls or text messages to obtain feedback on the vaccination consultation.

### 4.2. Consulting Preferences between HCWs and Residents 

Both HCWs and residents prefer specialized consulting led by HCWs, accompanied by follow-up through telephone. Compare those findings with studies that have examined the importance of specialized content in healthcare consultations, especially in the context of vaccine hesitancy. A community-based intervention in the United States addressed the knowledge and confidence gap regarding COVID-19 vaccination by employing branching logic and customizing content based on the user’s geographical location, rural–urban classification, race/ethnicity, and access to child-age-specific information [30]. Additionally, Osaghae’s research has demonstrated that parents exhibiting vaccine hesitancy were 61% more inclined to consistently receive HPV vaccination following follow-up counseling than their initial counseling session, further substantiating the significance of post-counseling interventions [31]. This suggests that during the training of HCWs in community health service centers, emphasis should be placed on cultivating the awareness of HCWs to provide consulting services and the flexibility of consultation proactively. The findings revealed that HCWs should provide more tailored recommendations based on individual patient circumstances rather than following a standardized approach [32]. However, there are also differences in consulting preferences between HCWs and residents. Residents prefer face-to-face consultation at community hospitals rather than online consultation, whereas HCWs do not express a specific preference for the consultation mode. In addition, for residents, the most essential attributes of consulting services are being consulting-led, followed by follow-up and content. With these aspects considered, the mode of consultation becomes less critical. However, for HCWs, the most crucial attribute of consulting services is a follow-up, followed by consulting mode and consulting-led.

The latent regression analysis in this study provides insights into the consulting preferences of individuals with chronic diseases and higher education levels. Individuals with chronic diseases and higher education levels may lean towards longer face-to-face traditional consulting sessions with specialized content. Conversely, healthy individuals and those with lower education levels may prefer shorter online standardized media consulting sessions (e.g., meeting their needs through WeChat public accounts). The underlying reason for this difference may lie in the more vital self-health awareness among vulnerable populations and those with higher education levels. They may seek tailored information to help weigh the benefits and risks, increasing self-efficacy and supporting reliable health decision-making [33]. Vulnerable populations, such as the elderly and individuals with chronic diseases, are often prioritized for multiple vaccines, including COVID-19 and influenza vaccines. In addition, patients afflicted with specific pathologies frequently seek extended direct consultations, driven by a requisite for detailed elucidation concerning the disease trajectory, therapeutic measures, and possible chronic sequelae [34]. Furthermore, individuals with a higher educational background often possess enhanced medical literacy, enabling more efficacious dialogues with healthcare professionals regarding their clinical status [35]; however, they are more likely to hesitate regarding vaccine acceptance due to their lower perception of vaccine safety influenced by their health-related factors. Paradoxically, vulnerable populations also face higher susceptibility and critical risks [36,37]. Therefore, improving vaccine coverage among vulnerable populations becomes a key focus of future health and immunization policies [38]. The exploratory results of this study on counseling preferences for residents with self-reported diseases could provide a scientific basis for future targeted policymaking. However, it is noted that this result has not reached statistical significance, possibly due to a small sample size, so more evidence and further expanded studies are called for validation.

From the resident’s perspective, the disparities in medical services between urban and rural areas are worth providing attention to. This study found that HCWs in urban areas prefer longer consultations with follow-up, while HCWs in rural areas show the opposite preference. In China, township health centers serve as the frontline connection between patients and the healthcare system [39,40]. However, rural primary healthcare faces many challenges, including a lack of qualified HCWs, poor service quality, and outdated medical facilities. Additionally, many rural residents are elderly with lower education levels, indicating that rural doctors must invest more time and patience. This contradicts the preferences observed in this study for rural doctors. Furthermore, intelligent follow-up modes through the internet, such as regular smartphone calls or text messages, can help establish better connections between residents and healthcare institutions [41,42]. However, due to the uneven distribution of healthcare consultation between urban and rural areas, this follow-up mode may not be prevalent in rural areas, contributing to the preference of rural doctors not to prioritize follow-up.

### 4.3. Research Prospects and Limitations

Based on the findings above and discussions, vaccination campaigns and strategies can implement impact measures to address the diverse preferences of HCWs and residents with varying health statuses in rural and urban areas for vaccination consultation methods. This is particularly significant for governments actively responding to global vaccine hesitancy.

Motivation strategy in rural areas: Considering the preference of rural doctors for efficient and concise face-to-face communication, vaccination strategies should prioritize the enhancement of on site education and individual consultation. For instance, organizing small-scale and brief community lectures for in-person verbal education, during which local doctors directly elucidate vaccines’ significance, safety, and efficacy while addressing residents’ inquiries. This intimate and direct communication may prove more effective in fostering trust and acceptance among rural residents.

Motivation strategy in urban areas: In light of the preference for online consultation and long-term follow-up among urban HCWs and some healthy residents, vaccination campaigns can leverage digital platforms and social media. This may involve developing an online system, providing real-time online consultation services, disseminating health education content, and implementing a feedback system to monitor changes in behavior and intentions among community residents. Health education can utilize concise videos, visual aids, and live content streaming to facilitate rapid and intuitive dissemination of information. This approach delivers fundamental knowledge about vaccines and promptly addresses public inquiries, particularly for urban HCWs who favor large-scale centralized management and are accustomed to digital interaction. 

Motivation strategy for residents with underlying conditions: This demographic may hesitate to get vaccinated due to concerns regarding potential vaccine side effects. Therefore, it is imperative to offer them comprehensive and long-term face-to-face consultations. By establishing dedicated consultation hours or organizing educational sessions on vaccination, they can engage in thorough discussions with healthcare professionals about the correlation between their health status and vaccination, ultimately alleviating their apprehension and fostering a greater willingness to receive vaccinations.

Based on the vaccination strategies above, the promotion campaign has the potential to significantly bolster trust, strategically impact vaccine hesitancy, and sustainably enhance community vaccination coverage. Employing communication methods preferred by healthcare workers can cultivate patient confidence in vaccination, elevate their engagement and satisfaction with the process, and augment resident vaccination rates through more effective information dissemination and resolution of doubts, mitigating vaccine hesitancy. Appropriate consulting programs are crucial for changing people’s perspectives, knowledge, and concerns regarding vaccine immunization [43]. The purpose of vaccination consultation is not to advise, persuade, or even instruct but rather to encourage better and more conscious vaccine awareness and greater acceptance. Engaging hesitant individuals through tailored communication strategies has been emphasized to alleviate their reluctance [44]. HCWs play a vital role in vaccine consultation, whether dealing with receptive or hesitant/unwilling residents [45]. Additionally, spreading misinformation in the self-media environment may be faster than transmitting pathogens. Consulting behavior can correct misconceptions among consultees promptly and convey more scientific and accurate information [46].

This study still has limitations. Firstly, although this study did not investigate the professional competence of individual HCWs, the perspectives of HCWs and other relevant personnel are crucial as they can influence residents’ professional identity and job motivation, thereby affecting their consulting behavior [47,48]. Secondly, this study exhibited a relatively high failure rate in the consistency test, which can be attributed to the extensive reading required for the discrete choice experiment and subsequent decision-making. It can be challenging for indecisive individuals to make choices at times, and only individuals who passed the consistency test were retained in the analysis. Thirdly, non-random sampling may result in selection bias, thereby impacting the representativeness of the population. Lastly, this study did not yield statistically significant results when comparing the preference categories of residents; however, education level and self-reported illness were close to the significance threshold and held practical value, as explained in the findings. Therefore, further research with larger sample sizes is anticipated to validate these results.

## 5. Conclusions

Vaccination consultation with HCWs is crucial in providing accurate information on vaccines to the general population. The selection preference for vaccine consultation reveals a gap between providers and demanders, with different groups exhibiting distinct preferences. For instance, rural HCWs prioritize efficient consultations, while their urban counterparts value long-term consultations, and vulnerable residents may lean towards traditional consultations. This study reaffirmed that consultees favor tailored vaccination consultation, which could accurately contribute to improving intervention acceptance. Thus, one of the priorities for health agencies is to specify a targeted counseling strategy for residents and HCWs with different characteristics and preferences. Identifying these targeted gaps can help design more acceptable and efficient interventions, increasing their likelihood of success and leading to better resource allocation for policymakers to develop targeted vaccination policies.

## Figures and Tables

**Table 1:** The characteristics of community residents and healthcare workers (HCWs).

Variable	Total		Consistency	
	n	Proportion (%)	n	Proportion (%)
**Residents**				
Overall	282	100	223	100
Age				
18–25 years old	63	22.3	54	24.2
26–35 years old	146	51.8	116	52
36+ years old	73	25.9	53	23.8
Sex				
Male	104	36.9	83	37.2
Female	178	63.1	140	62.8
Education				
High school and below	69	24.5	48	21.5
Bachelor and above	213	75.5	175	78.5
Monthly income ^a^				
RMB 0–5000	199	70.6	152	68.2
RMB >5000	83	29.4	71	31.8
Self-reported disease				
No	253	89.7	203	91
Yes	29	10.3	20	9
**Healthcare workers**				
Overall	204	100	158	100
Work age				
0–10 years	84	41.2	61	38.6
11–20 years	60	29.4	47	29.7
21–40 years	60	29.4	50	31.6
Sex				
Male	70	34.3	54	34.2
Female	134	65.7	104	65.8
Education				
High school and below	46	22.5	37	23.4
Bachelor and above	158	77.5	121	76.6
Workplace ^b^				
Rural	93	54.4	50	31.6
Urban	111	45.6	108	68.4
Professional title				
Primary	128	62.7	99	62.7
Middle and high	76	37.3	59	37.3

^a^ Renminbi (RMB) is the legal currency of the People’s Republic of China, RMB 1 ≈ USD 0.14. ^b^ The workplaces of HCWs were classified as either rural or urban by considering whether they worked in an urban community health service center or a rural township health center.

## Data Availability

All data to reproduce the results in the manuscript are available from the corresponding author upon reasonable request.

## Supplementary Materials

The following supporting information can be downloaded at: https://www.mdpi.com/article/10.3390/vaccines12050534/s1, Supplementary S1: Residents Questionnaire; Supplementary S2: HCWs Questionnaire; Supplementary S3: The consistency test of mixed-effects models in demanders; Supplementary S4: The consistency test of mixed-effects models in suppliers.

## References

[1] MacDonald N.E., SAGE Working Group on Vaccine Hesitancy (2015). Vaccine Hesitancy: Definition, Scope and Determinants. Vaccine.

[2] Costantino C., Caracci F., Brandi M., Bono S.E., Ferro A., Sannasardo C.E., Scarpitta F., Siddu A., Vella C., Ventura G. (2020). Determinants of Vaccine Hesitancy and Effectiveness of Vaccination Counseling Interventions among a Sample of the General Population in Palermo, Italy. Hum. Vaccin. Immunother..

[3] Yaqub O., Castle-Clarke S., Sevdalis N., Chataway J. (2014). Attitudes to Vaccination: A Critical Review. Soc. Sci. Med..

[4] Ramukumba M.M. (2020). Exploration of Community Health Workers’ Views about in Their Role and Support in Primary Health Care in Northern Cape, South Africa. J. Community Health.

[5] Nguyen K.H., Yankey D., Lu P.-J., Kriss J.L., Brewer N.T., Razzaghi H., Meghani M., Manns B.J., Lee J.T., Singleton J.A. (2021). Report of Health Care Provider Recommendation for COVID-19 Vaccination among Adults, by Recipient COVID-19 Vaccination Status and Attitudes—United States, April–September 2021. MMWR Morb. Mortal. Wkly Rep..

[6] Karlsson L.C., Lewandowsky S., Antfolk J., Salo P., Lindfelt M., Oksanen T., Kivimäki M., Soveri A. (2019). The Association between Vaccination Confidence, Vaccination Behavior, and Willingness to Recommend Vaccines among Finnish Healthcare Workers. PLoS ONE.

[7] Diks M.E., Hiligsmann M., van der Putten I.M. (2021). Vaccine Preferences Driving Vaccine-Decision Making of Different Target Groups: A Systematic Review of Choice-Based Experiments. BMC Infect. Dis..

[8] Díaz Luévano C., Sicsic J., Pellissier G., Chyderiotis S., Arwidson P., Olivier C., Gagneux-Brunon A., Botelho-Nevers E., Bouvet E., Mueller J. (2021). Quantifying Healthcare and Welfare Sector Workers’ Preferences around COVID-19 Vaccination: A Cross-Sectional, Single-Profile Discrete-Choice Experiment in France. BMJ Open.

[9] Lancsar E., Fiebig D.G., Hole A.R. (2017). Discrete Choice Experiments: A Guide to Model Specification, Estimation and Software. Pharmacoeconomics.

[10] Soekhai V., de Bekker-Grob E.W., Ellis A.R., Vass C.M. (2019). Discrete Choice Experiments in Health Economics: Past, Present and Future. Pharmacoeconomics.

[11] Arora N., Crastes Dit Sourd R., Hanson K., Woldesenbet D., Seifu A., Quaife M. (2022). Linking Health Worker Motivation with Their Stated Job Preferences: A Hybrid Choice Analysis in Ethiopia. Soc. Sci. Med..

[12] Karim S., Craig B.M., Vass C., Groothuis-Oudshoorn C.G.M. (2022). Current Practices for Accounting for Preference Heterogeneity in Health-Related Discrete Choice Experiments: A Systematic Review. Pharmacoeconomics.

[13] Lamba S., Arora N., Keraga D.W., Kiflie A., Jembere B.M., Berhanu D., Dubale M., Marchant T., Schellenberg J., Umar N. (2021). Stated Job Preferences of Three Health Worker Cadres in Ethiopia: A Discrete Choice Experiment. Health Policy Plan..

[14] Bhat C.R. (1997). An Endogenous Segmentation Mode Choice Model with an Application to Intercity Travel. Transp. Sci..

[15] Train K.E. (2008). EM Algorithms for Nonparametric Estimation of Mixing Distributions. J. Choice Model..

[16] Januszek S., Siwiec N., Januszek R., Kluz M., Lebed R., Toś P., Góra T., Plens K., Dąbrowski K., Sidorowicz M. (2022). Approach of Pregnant Women from Poland and the Ukraine to COVID-19 Vaccination—The Role of Medical Consultation. Vaccines.

[17] Prematunge C., Corace K., McCarthy A., Nair R.C., Pugsley R., Garber G. (2012). Factors Influencing Pandemic Influenza Vaccination of Healthcare Workers—A Systematic Review. Vaccine.

[18] Ceulemans M., Foulon V., Panchaud A., Winterfeld U., Pomar L., Lambelet V., Cleary B., O’Shaughnessy F., Passier A., Richardson J.L. (2021). Vaccine Willingness and Impact of the COVID-19 Pandemic on Women’s Perinatal Experiences and Practices-A Multinational, Cross-Sectional Study Covering the First Wave of the Pandemic. Int. J. Environ. Res. Public Health.

[19] Skjefte M., Ngirbabul M., Akeju O., Escudero D., Hernandez-Diaz S., Wyszynski D.F., Wu J.W. (2021). COVID-19 Vaccine Acceptance among Pregnant Women and Mothers of Young Children: Results of a Survey in 16 Countries. Eur. J. Epidemiol..

[20] Wang K., Wong E.L.-Y., Cheung A.W.-L., Dong D., Yeoh E.-K. (2023). Loss-Framing of Information and Pre-Vaccination Consultation Improve COVID-19 Vaccine Acceptance: A Survey Experiment. Front. Public. Health.

[21] Tianshuo Z., Hanyu L., Bingfeng H., Bei L., Jiang L., Juan D., Ninghua H., Qingbin L., Yaqiong L., Fuqiang C. (2022). Evaluation of the Reliability and Validity of a Vaccine Hesitancy Scale on Knowledge, Attitude, Trust and Vaccination Environment (KATE-S) in Chinese Parents. Vaccine.

[22] Kasting M.L., Macy J.T., Grannis S.J., Wiensch A.J., Lavista Ferres J.M., Dixon B.E. (2022). Factors Associated With the Intention to Receive the COVID-19 Vaccine: Cross-Sectional National Study. JMIR Public Health Surveill..

[23] Volpp K.G., Loewenstein G., Buttenheim A.M. (2021). Behaviorally Informed Strategies for a National COVID-19 Vaccine Promotion Program. JAMA.

[24] Wang K., Wong E.L.-Y., Ho K.-F., Cheung A.W.-L., Yau P.S.-Y., Dong D., Wong S.Y.-S., Yeoh E.-K. (2021). Change of Willingness to Accept COVID-19 Vaccine and Reasons of Vaccine Hesitancy of Working People at Different Waves of Local Epidemic in Hong Kong, China: Repeated Cross-Sectional Surveys. Vaccines.

[25] Mazzi M.A., Rimondini M., van der Zee E., Boerma W., Zimmermann C., Bensing J. (2018). Which Patient and Doctor Behaviours Make a Medical Consultation More Effective from a Patient Point of View. Results from a European Multicentre Study in 31 Countries. Patient Educ. Couns..

[26] Rodríguez Torres A., Jarillo Soto E.C., Casas Patiño D. (2018). Medical consultation, time and duration. Medwave.

[27] Derse A.R. (2022). The Physician-Patient Relationship. N. Engl. J. Med..

[28] Ma X., Lu J., Liu W. (2021). Influencing Factors on Health Information to Improve Public Health Literacy in the Official WeChat Account of Guangzhou CDC. Front. Public Health.

[29] Sun M., Yang L., Chen W., Luo H., Zheng K., Zhang Y., Lian T., Yang Y., Ni J. (2021). Current Status of Official WeChat Accounts for Public Health Education. J. Public Health.

[30] McCulloh R.J., Darden P.M., Snowden J., Ounpraseuth S., Lee J., Clarke M., Newcomer S.R., Fu L., Hubberd D., Baldner J. (2022). Improving Pediatric COVID-19 Vaccine Uptake Using an mHealth Tool (MoVeUp): Study Protocol for a Randomized, Controlled Trial. Trials.

[31] Osaghae I., Darkoh C., Chido-Amajuoyi O.G., Chan W., Padgett Wermuth P., Pande M., Cunningham S.A., Shete S. (2023). Healthcare Provider’s Perceived Self-Efficacy in HPV Vaccination Hesitancy Counseling and HPV Vaccination Acceptance. Vaccines.

[32] Omer S.B., Benjamin R.M., Brewer N.T., Buttenheim A.M., Callaghan T., Caplan A., Carpiano R.M., Clinton C., DiResta R., Elharake J.A. (2021). Promoting COVID-19 Vaccine Acceptance: Recommendations from the Lancet Commission on Vaccine Refusal, Acceptance, and Demand in the USA. Lancet.

[33] Chen Y., Gu W., He B., Gao H., Sun P., Li Q., Chen E., Miao Z. (2022). Impact of a Community-Based Health Education Intervention on Awareness of Influenza, Pneumonia, and Vaccination Intention in Chronic Patients. Hum. Vaccin. Immunother..

[34] Gong X., Hou M., Guo R., Feng X.L. (2022). Investigating the Relationship between Consultation Length and Quality of Tele-Dermatology E-Consults in China: A Cross-Sectional Standardized Patient Study. BMC Health Serv. Res..

[35] Jansen T., Rademakers J., Waverijn G., Verheij R., Osborne R., Heijmans M. (2018). The Role of Health Literacy in Explaining the Association between Educational Attainment and the Use of Out-of-Hours Primary Care Services in Chronically Ill People: A Survey Study. BMC Health Serv. Res..

[36] Wong M.C.S., Wong E.L.Y., Huang J., Cheung A.W.L., Law K., Chong M.K.C., Ng R.W.Y., Lai C.K.C., Boon S.S., Lau J.T.F. (2021). Acceptance of the COVID-19 Vaccine Based on the Health Belief Model: A Population-Based Survey in Hong Kong. Vaccine.

[37] Wang G., Yao Y., Wang Y., Gong J., Meng Q., Wang H., Wang W., Chen X., Zhao Y. (2023). Determinants of COVID-19 Vaccination Status and Hesitancy among Older Adults in China. Nat. Med..

[38] Doherty M., Schmidt-Ott R., Santos J.I., Stanberry L.R., Hofstetter A.M., Rosenthal S.L., Cunningham A.L. (2016). Vaccination of Special Populations: Protecting the Vulnerable. Vaccine.

[39] Yang H., Han J., Xu Y., Gao X., Wang Y., Yang Y., Cao X. (2022). Ten years of development of China’s general practice industry: Opportunities and challenges coexist. Chin. Gen. Pract..

[40] Gu L., Tian B., Xin Y., Zhang S., Li J., Sun Z. (2022). Patient Perception of Doctor Communication Skills and Patient Trust in Rural Primary Health Care: The Mediating Role of Health Service Quality. BMC Prim. Care.

[41] Pan X., Zhou X., Yu L., Hou L. (2023). Switching from Offline to Online Health Consultation in the Post-Pandemic Era: The Role of Perceived Pandemic Risk. Front. Public Health.

[42] Li X., Jin Y. (2023). The development prospect of Internet + nursing service model. Health Vocat. Educ..

[43] Kaufman J., Ryan R., Walsh L., Horey D., Leask J., Robinson P., Hill S. (2018). Face-to-Face Interventions for Informing or Educating Parents about Early Childhood Vaccination. Cochrane Database Syst. Rev..

[44] MacDonald N.E., Desai S., Gerstein B. (2018). Working with Vaccine-Hesitant Parents: An Update. Paediatr. Child. Health.

[45] Jarrett C., Wilson R., O’Leary M., Eckersberger E., Larson H.J., SAGE Working Group on Vaccine Hesitancy (2015). Strategies for Addressing Vaccine Hesitancy—A Systematic Review. Vaccine.

[46] Altmann D.M., Boyton R.J. (2022). COVID-19 Vaccination: The Road Ahead. Science.

[47] Pal S., Shekhar R., Kottewar S., Upadhyay S., Singh M., Pathak D., Kapuria D., Barrett E., Sheikh A.B. (2021). COVID-19 Vaccine Hesitancy and Attitude toward Booster Doses among US Healthcare Workers. Vaccines.

[48] Dzieciolowska S., Hamel D., Gadio S., Dionne M., Gagnon D., Robitaille L., Cook E., Caron I., Talib A., Parkes L. (2021). COVID-19 Vaccine Acceptance, Hesitancy, and Refusal among Canadian Healthcare Workers: A Multicenter Survey. Am. J. Infect. Control.

