# Supplementary material for: Differences in Vaccination Consultation Preferred by Primary Health Care Workers and Residents in Community Settings
Source: Vaccines (Basel). 2024 May 14;12(5):534. doi: 10.3390/vaccines12050534 (PMC11126119; doi:10.3390/vaccines12050534)
Supplement: Supplementary file 1 [file vaccines-12-00534-s001.zip › vaccines-2960087-SI.pdf]

Supplementary S1. Residents Questionnaire

I. Socio-demographic information

1. What is your gender?
   a) male
   b) female
2. What is your age? ______years old
3. What is your highest educational level?
   a) high school and below
   b) junior college and above
4. What is your average monthly income?
   a) less than 5,000 RMB
   b) more than 5,000 RMB
5. Do you combine the following health conditions [multiple choices]:
   a) no
   b) obesity (BMI>30)
   c) hypertension
   d) hyperlipidemia
   e) diabetes
   f) cardiovascular and cerebrovascular diseases
   g) chronic lung diseases (such as COPD, chronic pneumonia, etc.)
   h) chronic liver diseases (such as chronic hepatitis, cirrhosis, etc.)
   i) chronic kidney disease or neoplasm
   j) tumor
   k) immune dysfunction (e.g., AIDS)
   l) other underlying disease
6. What are your main considerations for vaccination or non-vaccination? Please select the three **most important and rank** them in the order from most important to least important, and fill in the number below: (1) (2) _(3) _
   a) effectiveness of vaccine
   b) safety of vaccine
   c) severity of disease
   d) susceptibility of disease
   e) media coverage
   f) time taken
   g) community (or affiliated unit) requirements
   h) financial rewards
   i) advice from others
   j) vaccination behavior from others
   k) techniques and attitudes of vaccinators
   l) vaccination experience

Instructions: There will be several exclusive choice questions, each asking you to choose which of two vaccination advice services supplied by health care workers (HCWs) you prefer, you can also choose "Neither" when you really can't make a choice.

7. If the HCWs can provide you with vaccination-related consultation services, which of the following modes do you prefer to use for consultation?
   a) Consultation mode A
   b) Consultation mode B
   c) Neither

Attribute	Consulting Mode A	Counseling Mode B
**Consultation-led**	Demander (HCWs begin offering counseling services after patients ask about it)	Supplier (HCWs take the initiative to ask about vaccination status and offer counseling services)
**Consulting duration**	<5 minutes	>5 minutes
**Consulting content**	Specialized (HCWs provide targeted consultations according to the characteristics and needs of residents)	Normalized (HCWs conduct comprehensive but not targeted consultations according to the guidelines)
**Consulting mode**	Online (e.g. new media or instant messaging)	Offline (Face-to-face interviews)
**Telephone follow-up**	Without follow-up visits	With follow-up visits

8. If the HCWs can provide you with vaccination-related consultation services, which of the following modes do you prefer to use for consultation?
   a) Consultation mode A
   b) Consultation mode B
   c) Neither

Attribute	Consulting Mode A	Counseling Mode B
**Consultation-led**	Demander (HCWs begin offering counseling services after patients ask about it)	Supplier (HCWs take the initiative to ask about vaccination status and offer counseling services) counseling services
**Consulting duration**	>5 minutes	<5 minutes
**Consulting content**	Specialized (HCWs provide targeted consultations according to the characteristics and needs of residents)	Normalized (HCWs conduct comprehensive but not targeted consultations according to the guidelines) guidelines)
**Consulting mode**	Online (e.g. new media or instant messaging)	Offline (Face-to-face interviews)
**Telephone follow-up**	Without follow-up visits	With follow-up visits

9. If the HCWs can provide you with vaccination-related consultation services, which of the following modes do you prefer to use for consultation?

a) Consultation mode A
b) Consultation mode B
c) Neither

Attribute	Consulting Mode A	Counseling Mode B
**Consultation-led**	Demander (HCWs begin offering counseling services after patients ask about it)	Supplier (HCWs take the initiative to ask about vaccination status and offer counseling services)
**Consulting duration**	<5 minutes	>5 minutes
**Consulting content**	Specialized (HCWs provide targeted consultations according to the characteristics and needs of residents	Normalized (HCWs conduct comprehensive but not targeted consultations according to the guidelines)
**Consulting mode**	Offline (Face-to-face interviews)	Online (e.g. new media or instant messaging)
**Telephone follow-up**	Without follow-up visits	With follow-up visits

10. If the HCWs can provide you with vaccination-related consultation services, which of the following modes do you prefer to use for consultation?
a) Consultation mode A
b) Consultation mode B
c) Neither

Attribute	Consulting Mode A	Counseling Mode B
**Consultation-led**	Demander (HCWs begin offering counseling services after patients ask about it)	Supplier (HCWs take the initiative to ask about vaccination status and offer counseling services)
**Consulting duration**	>5 minutes	<5 minutes
**Consulting content**	Specialized (HCWs provide targeted consultations according to the characteristics and needs of residents)	Normalized (HCWs conduct comprehensive but not targeted consultations according to the guidelines)
**Consulting mode**	Online (e.g. new media or instant messaging)	Offline (Face-to-face interviews)
**Telephone follow-up**	With follow-up visits	Without follow-up visits

11. If the HCWs can provide you with vaccination-related consultation services, which of the following modes do you prefer to use for consultation?
a) Consultation mode A
b) Consultation mode B
c) Neither

Attribute	Consulting Mode A	Counseling Mode B

**Consultation-led**	Demander (HCWs begin offering counseling services after patients ask about it)	Supplier (HCWs take the initiative to ask about vaccination status and offer counseling services)
**Consulting duration**	<5 minutes	>5 minutes
**Consulting content**	Normalized (HCWs conduct comprehensive but not targeted consultations according to the guidelines)	Specialized (HCWs provide targeted consultations according to the characteristics and needs of residents)
**Consulting mode**	Online (e.g. new media or instant messaging)	Offline (Face-to-face interviews)
**Telephone follow-up**	With follow-up visits	Without follow-up visits

12. If the HCWs can provide you with vaccination-related consultation services, which of the following modes do you prefer to use for consultation?
    a) Consultation mode A
    b) Consultation mode B
    c) Neither

Attribute	Consulting Mode A	Counseling Mode B
**Consultation-led**	Demander (HCWs begin offering counseling services after patients ask about it)	Supplier (HCWs take the initiative to ask about vaccination status and offer counseling services)
**Consulting duration**	>5 minutes	<5 minutes
**Consulting content**	Specialized (HCWs provide targeted consultations according to the characteristics and needs of residents)	Normalized (HCWs conduct comprehensive but not targeted consultations according to the guidelines)
**Consulting mode**	Offline (Face-to-face interviews)	Online (e.g. new media or instant messaging)
**Telephone follow-up**	With follow-up visits	Without follow-up visits

13. If the HCWs can provide you with vaccination-related consultation services, which of the following modes do you prefer to use for consultation?
    a) Consultation mode A
    b) Consultation mode B
    c) Neither

Attribute	Consulting Mode A	Counseling Mode B
**Consultation-led**	Demander (HCWs begin offering counseling services after patients ask about it)	Supplier (HCWs take the initiative to ask about vaccination status and offer counseling services)

**Consulting duration**	<5 minutes	>5 minutes
**Consulting content**	Specialized (HCWs provide targeted consultations according to the characteristics and needs of residents)	Normalized (HCWs conduct comprehensive but not targeted consultations according to the guidelines)
**Consulting mode**	Offline (Face-to-face interviews)	Online (e.g. new media or instant messaging)
**Telephone follow-up**	With follow-up visits	Without follow-up visits

14. If the HCWs can provide you with vaccination-related consultation services, which of the following modes do you prefer to use for consultation?
    a) Consultation mode A
    b) Consultation mode B
    c) Neither

Attribute	Consulting Mode A	Counseling Mode B
**Consultation-led**	Demander (HCWs begin offering counseling services after patients ask about it)	Supplier (HCWs take the initiative to ask about vaccination status and offer counseling services)
**Consulting duration**	>5 minutes	<5 minutes
**Consulting content**	Normalized (HCWs conduct comprehensive but not targeted consultations according to the guidelines)	Specialized (HCWs provide targeted consultations according to the characteristics and needs of residents)
**Consulting mode**	Offline (Face-to-face interviews)	Online (e.g. new media or instant messaging)
**Telephone follow-up**	Without follow-up visits	With follow-up visits

Supplementary S2. HCWs Questionnaire

I. Baseline information

1. What is your gender?
   a) male
   b) female
2. What is your work age? ________years
3. What is your highest educational level?
   a) high school and below
   b) junior college and above
4. What is your professional title?
   a) primary
   b) middle
   c) high
5. What is your work address?
   _______________city ___________district (county) _____________community (township)
6. Which of the following do you think can facilitate you to provide vaccination advice or vaccination consultation services to patients/residents? Please select the three most important ones and **rank** them in the order of most important → least important, and fill in the number below
   28. (1) __________(2) __________(3) __________
   a) skill training
   b) consulting guidelines,
   c) vaccination guidelines (more specific)
   d) subsidies
   e) honorary certificates
   f) opportunities for further education

Instructions: There will be several exclusive choice questions, each asking you to choose which of two vaccination advice services demanded by residents you prefer, you can also choose "Neither" when you really can't make a choice.

7. If you can provide residents/patients with vaccination-related consultation services, which of the following modes do you prefer to use for consultation?
   a) Consultation mode A
   b) Consultation mode B
   c) Neither

Attribute	Consulting Mode A	Counseling Mode B
**Consultation-led**	Demander (HCWs begin offering counseling services after patients ask about it)	Supplier (HCWs take the initiative to ask about vaccination status and offer counseling services)
**Consulting duration**	<5 minutes	>5 minutes
**Consulting content**	Specialized (HCWs provide targeted consultations according to the characteristics and needs of residents)	Normalized (HCWs conduct comprehensive but not targeted consultations according to the guidelines)
**Consulting mode**	Online (e.g. new media or instant messaging)	Offline (Face-to-face interviews)
**Telephone follow-up**	Without follow-up visits	With follow-up visits

8. If you can provide residents/patients with vaccination-related consultation services, which of the following modes do you prefer to use for consultation?
   a) Consultation mode A
   b) Consultation mode B
   c) Neither

Attribute	Consulting Mode A	Counseling Mode B
**Consultation-led**	Demander (HCWs begin offering counseling services after patients ask about it)	Supplier (HCWs take the initiative to ask about vaccination status and offer counseling services)
**Consulting duration**	>5 minutes	<5 minutes
**Consulting content**	Specialized (HCWs provide targeted consultations according	Normalized (HCWs conduct comprehensive but not

	to the characteristics and needs of residents)	targeted consultations according to the guidelines)
**Consulting mode**	Online (e.g. new media or instant messaging)	Offline (Face-to-face interviews)
**Telephone follow-up**	Without follow-up visits	With follow-up visits

9. If you can provide residents/patients with vaccination-related consultation services, which of the following modes do you prefer to use for consultation?
   a) Consultation mode A
   b) Consultation mode B
   c) Neither

**Attribute**	**Consulting Mode A**	**Counseling Mode B**
**Consultation-led**	Demander (HCWs begin offering counseling services after patients ask about it)	Supplier (HCWs take the initiative to ask about vaccination status and offer counseling services)
**Consulting duration**	<5 minutes	>5 minutes
**Consulting content**	Specialized (HCWs provide targeted consultations according to the characteristics and needs of residents	Normalized (HCWs conduct comprehensive but not targeted consultations according to the guidelines)
**Consulting mode**	Offline (Face-to-face interviews)	Online (e.g. new media or instant messaging)
**Telephone follow-up**	Without follow-up visits	With follow-up visits

10. If you can provide residents/patients with vaccination-related consultation services, which of the following modes do you prefer to use for consultation?
   a) Consultation mode A
   b) Consultation mode B
   c) Neither

**Attribute**	**Consulting Mode A**	**Counseling Mode B**

**Consultation-led**	Demander (HCWs begin offering counseling services after patients ask about it)	Supplier (HCWs take the initiative to ask about vaccination status and offer counseling services)
**Consulting duration**	>5 minutes	<5 minutes
**Consulting content**	Specialized (HCWs provide targeted consultations according to the characteristics and needs of residents)	Normalized (HCWs conduct comprehensive but not targeted consultations according to the guidelines)
**Consulting mode**	Online (e.g. new media or instant messaging)	Offline (Face-to-face interviews)
**Telephone follow-up**	With follow-up visits	Without follow-up visits

11. If you can provide residents/patients with vaccination-related consultation services, which of the following modes do you prefer to use for consultation?
    a) Consultation mode A
    b) Consultation mode B
    c) Neither

Attribute	Consulting Mode A	Counseling Mode B
**Consultation-led**	Demander (HCWs begin offering counseling services after patients ask about it)	Supplier (HCWs take the initiative to ask about vaccination status and offer counseling services)
**Consulting duration**	<5 minutes	>5 minutes
**Consulting content**	Normalized (HCWs conduct comprehensive but not targeted consultations according to the guidelines)	Specialized (HCWs provide targeted consultations according to the characteristics and needs of residents)
**Consulting mode**	Online (e.g. new media or instant messaging)	Offline (Face-to-face interviews)
**Telephone follow-up**	With follow-up visits	Without follow-up visits

12. If you can provide residents/patients with vaccination-related consultation services, which of the

following modes do you prefer to use for consultation?
a) Consultation mode A
b) Consultation mode B
c) Neither

Attribute	Consulting Mode A	Counseling Mode B
**Consultation-led**	Demander (HCWs begin offering counseling services after patients ask about it)	Supplier (HCWs take the initiative to ask about vaccination status and offer counseling services)
**Consulting duration**	>5 minutes	<5 minutes
**Consulting content**	Specialized (HCWs provide targeted consultations according to the characteristics and needs of residents)	Normalized (HCWs conduct comprehensive but not targeted consultations according to the guidelines)
**Consulting mode**	Offline (Face-to-face interviews)	Online (e.g. new media or instant messaging)
**Telephone follow-up**	With follow-up visits	Without follow-up visits

13. If you can provide residents/patients with vaccination-related consultation services, which of the following modes do you prefer to use for consultation?
a) Consultation mode A
b) Consultation mode B
c) Neither

Attribute	Consulting Mode A	Counseling Mode B
**Consultation-led**	Demander (HCWs begin offering counseling services after patients ask about it)	Supplier (HCWs take the initiative to ask about vaccination status and offer counseling services)
**Consulting duration**	<5 minutes	>5 minutes
**Consulting content**	Specialized (HCWs provide targeted consultations according to the characteristics and needs of residents)	Normalized (HCWs conduct comprehensive but not targeted consultations according to the guidelines)

**Consulting mode**	Offline (Face-to-face interviews)	Online (e.g. new media or instant messaging)
**Telephone follow-up**	With follow-up visits	Without follow-up visits

14. If you can provide residents/patients with vaccination-related consultation services, which of the following modes do you prefer to use for consultation?
    a) Consultation mode A
    b) Consultation mode B
    c) Neither

Attribute	Consulting Mode A	Counseling Mode B
**Consultation-led**	Demander (HCWs begin offering counseling services after patients ask about it)	Supplier (HCWs take the initiative to ask about vaccination status and offer counseling services)
**Consulting duration**	>5 minutes	<5 minutes
**Consulting content**	Normalized (HCWs conduct comprehensive but not targeted consultations according to the guidelines)	Specialized (HCWs provide targeted consultations according to the characteristics and needs of residents)
**Consulting mode**	Offline (Face-to-face interviews)	Online (e.g. new media or instant messaging)
**Telephone follow-up**	Without follow-up visits	With follow-up visits

Supplementary S3. The consistency test of mixed-effects models in demanders.

Property (*Reference*)	Coefficient		SD		Log likelihood	AIC	BIC
	Mean (SE)	*P* value	Mean (SE)	*P* value			
Model 1							
Consulting-led (*Residents*)	1.954 (0.284)	<0.001	3.111 (0.405)	<0.001	-911.691	1843.381	1904.649
Consulting duration (<*5 min*)	-0.104 (0.193)	0.588	1.949 (0.351)	<0.001			
Consulting content (*Normalized*)	0.835 (0.235)	<0.001	-2.298 (0.300)	<0.001			
Consulting mode (*Online*)	0.151 (0.225)	0.503	2.691 (0.402)	<0.001			
Telephone follow-up (*No*)	1.123 (0.194)	<0.001	1.973 (0.304)	<0.001			
Model 2							
Consulting-led (*Residents*)	2.168 (0.375)	<0.001	3.570 (0.511)	<0.001	-689.931	1399.861	1458.782
Consulting duration (<*5 min*)	0.207 (0.223)	0.353	-1.540 (0.333)	<0.001			
Consulting content (*Normalized*)	0.954 (0.303)	0.002	3.965 (0.544)	<0.001			
Consulting mode (*Online*)	0.462 (0.267)	0.083	2.812 (0.408)	<0.001			
Telephone follow-up (*No*)	1.552 (0.300)	<0.001	2.231 (0.389)	<0.001			

Note: Model 1, mixed effect models of all include demanders; Model 2, mixed effects model of the demanders who passed the consistency test. The likelihood-ratio test was used to verify the hypothesis that Model 1 nested within Model 2, and the result rejected the original hypothesis.

Supplementary S4. The consistency test of mixed-effects models in suppliers.

Property (*Reference*)	Coefficient		SD		Log likelihood	AIC	BIC
	Mean (SE)	*P* value	Mean (SE)	*P* value			
Model 1							
Consulting-led (*Patient*)	0.387 (0.155)	0.012	2.170 (0.190)	<0.001	-1411.590	2843.180	2908.142
Consulting duration (<*5 min*)	-0.019 (0.095)	0.841	-0.893 (0.132)	<0.001			
Consulting content (*Normalized*)	0.272 (0.100)	0.007	1.004 (0.131)	<0.001			
Consulting mode (*Online*)	0.492 (0.078)	<0.001	0.396 (0.144)	0.006			
Telephone follow-up (*No*)	0.873 (0.102)	<0.001	1.014 (0.124)	<0.001			
Model 2							
Consulting-led (*Patient*)	0.458 (0.224)	0.041	2.638 (0.257)	<0.001	-1051.089	2122.177	2184.584
Consulting duration (<*5 min*)	0.016 (0.112)	0.886	0.891 (0.143)	<0.001			
Consulting content (*Normalized*)	0.409 (0.122)	0.001	0.998 (0.140)	<0.001			
Consulting mode (*Online*)	0.540 (0.100)	<0.001	0.649 (0.152)	<0.001			
Telephone follow-up (*No*)	0.831 (0.126)	<0.001	1.222 (0.161)	<0.001			

Note: Model 1, mixed effect models of all include suppliers; Model 2, mixed effects model of the suppliers who passed the consistency test. The likelihood-ratio test was used to verify the hypothesis that Model 1 nested within Model 2, and the result rejected the original hypothesis.
